# Association of neurofascin IgG4 and atypical chronic inflammatory demyelinating polyneuropathy: A systematic review and meta‐analysis

**DOI:** 10.1002/brb3.1115

**Published:** 2018-09-21

**Authors:** Wenyu Hu, Yanguo Xin, Zhiyi He, Yinan Zhao

**Affiliations:** ^1^ Department of Cardiology the First Affiliated Hospital of China Medical University Shenyang China; ^2^ Department of Neurology the First Affiliated Hospital of China Medical University Shenyang China

**Keywords:** chronic inflammatory demyelinating polyradiculoneuropathy, diagnosis, meta‐analysis, NF155

## Abstract

**Background:**

Chronic inflammatory demyelinating polyradiculoneuropathy (CIDP) is the most commonly observed phenotype among chronic acquired demyelinating polyneuropathies and is clinically variable. The aim of this meta‐analysis was to evaluate the diagnostic value and characteristics of CIDP targeting neurofascin 155 (NF155).

**Methods:**

A systematic literature search was performed on March 2018, and two reviewers independently extracted data and assessed the risk of bias on MEDLINE, EMBASE, the Web of Science, and the Cochrane Library to identify relevant articles.

**Results:**

Ten articles for the NF155 protein test with 1,161 patients and 1,636 controls were identified. The results showed that the pooled sensitivity was 0.09 (95% CI: 0.06–015), and specificity was 1.00 (95% CI: 0.98–1.00) of the NF155 for CIDP. The meta‐analysis revealed that the sensory ataxic occurrence rate (OR: 10.79, 95% CI: 5.24–22.22) and tremor occurrence rate (OR: 6.71, 95% CI: 3.37–13.39) were higher among patients positive for NF155 compared with NF155‐negative CIDP patients. However, the rate of good treatment response to intravenous immunoglobulin (IVIg) (OR: 0.09, 95% CI: 0.02–0.42) was lower in NF155‐positive CIDP patients.

**Conclusions:**

NF155 is a specific protein marker for CIDP, but its diagnostic value has been questioned due to low sensitivity. However, as an antibody against paranodal antigens, NF155 seems more valuable in defining clinical subsets of CIDP.

## 1 INTRODUCTION

Chronic acquired demyelinating polyneuropathy is a group of autoimmune diseases involving the peripheral nerves, from which approximately 420,000 individuals worldwide are suffering. The most common phenotype, chronic inflammatory demyelinating polyradiculoneuropathy (CIDP), is clinically variable (Latov, 2014). CIDP has been clinically classified into “typical” or “atypical” cases (Van den Bergh et al., 2010). Typical CIDP is diagnosed as a symmetric motor/sensory dysfunction with proximal and distal weakness, flexia with conduction slowing, time dispersion, and/or conduction block in the electrophysiological examination (Hughes et al., 2006; Van den Bergh et al., 2010). Supporting diagnostic evidence includes spinal fluid albuminocytological dissociation, MRI‐based evidence of enlarged, enhancing nerve roots, and/or objective evidence of response to immune treatments (Hughes et al., 2006; Van den Bergh et al., 2010). Atypical CIDP patients typically show extensive heterogeneity in the clinical features and the response rate to treatments and can be difficult to clinically identify according to the electrophysiological criteria. Establishing a new method to distinguish the atypical CIDP from the typical cases will greatly facilitate accurate diagnoses and effective application of treatment options for atypical CIDP patients. Thus, the detection of specific biomarkers is not only crucial to identifying CIDP but also to distinguish subtypes and guide accurate treatment. Recently, several studies revealed that atypical CIDP is associated with anti‐NF155 (Devaux, Miura, Fukami, Inoue, Manso, & Belghazi, 2016; Kadoya, Kaida, Koike, Takazaki, Ogata, & Moriguchi, 2016; Mathey, Garg, Park, Nguyen, Baker, & Yuki, 2017; Ogata, Yamasaki, Hiwatashi, Oka, Kawamura, & Matsuse, 2015).

NF155 is a member of the L1 family of adhesion molecules; it is located at the paranode and expressed by the terminal loops of myelin. Together with the axonal cell adhesion molecules, CNTN‐1 and contactin‐associated protein‐1 (Caspr1), NF155 forms septate‐like junctions that anchor the myelin loops to the axon (Charles et al., 2002). Loss of the attachment changes the nodal architecture and exposes K^+^ channels in the juxtaparanodal region to limit saltatory conduction, which ultimately causes conduction block and deceleration. Since an initial report by Ng, et al. (2012), several studies have documented that immunoglobulin G4 (IgG4) autoantibodies to NF155 are observed in a small proportion of patients with CIDP (Burnor, Yang, Zhou, Patterson, Quinn, & Reilly, 2018; Devaux et al., 2016; Kadoya et al., 2016; Mathey et al., 2017; Ogata et al., 2015; Querol, Nogales‐Gadea, Rojas‐Garcia, Diaz‐Manera, Pardo, & Ortega‐Moreno, 2014). IgG4 anti‐NF155‐positive CIDP exhibits distinguished clinical features compared with IgG4 anti‐NF155‐negative ones, including specific immunotherapeutic response. IgG4 anti‐NF155 had been reported as the etiology of CIDP (Burnor et al., 2018; Devaux et al., 2016; Doppler, Appeltshauser, Kramer, Ng, Meinl, & Villmann, 2015; Kadoya et al., 2016; Kawamura, Yamasaki, Yonekawa, Matsushita, Kusunoki, & Nagayama, 2013; Mathey et al., 2017; Ng, J. Malotka, Kawakami, Derfuss, Khademi, & Olsson, 2012; Ogata et al., 2015; Querol et al., 2014; Yan, Nguyen, Yuki, Ji, Yiannikas, & Pollard, 2014); however, there are still several limitations in these studies to evaluate the diagnostic value of anti‐NF155 in CIDP patients. First, sample sizes have been small in every clinical study, and due to these small sample sizes, statistical correlations are doubtful. Second, discrepancy of the anti‐NF155 detection frequencies and different conclusions exists in these papers. Third, in addition to anti‐NF155, the diagnostic value of two other paranodal and nodal proteins, anti‐CNTN1 and anti‐NF186, also need to be evaluated. Fourth, more in‐depth discussion regarding the potential pathomechanism of the anti‐NF155 in CIDP is necessary. Therefore, we aimed to integrate all published evidence systematically in this meta‐analysis to discover the various roles of anti‐NF155, anti‐CNTN1, and anti‐NF186 in CIDP. We also reviewed the potential pathomechanism of anti‐NF155 in CIDP patients. We hope that our data can offer a more precise diagnostic and therapeutic value of anti‐NF155 to CIDP patients and inspire the readers to focus on the roles of other paranodal/nodal proteins, such as anti‐CNTN1 and anti‐NF186, in CIDP.

## 2 MATERIALS AND METHODS

### 2.1 Search strategy and study selection

This systematic literature review and meta‐analysis were performed using the methodology suggested by the Preferred Reporting Items for Systematic Reviews and Meta‐Analysis (PRISMA) guidelines. A systematic literature search was performed in English on March 2018 in the following databases: MEDLINE, PubMed, EMBASE, the Web of Science, and the Cochrane Library. The keywords used were the following: chronic inflammatory demyelinating polyradiculoneuropathy, chronic acquired demyelinating polyneuropathies, neurofascin, IgG4 autoantibodies, and NF155. The keywords were combined with appropriate Boolean operators, and for further relevant articles, we also checked the reference lists of all the identified trials. After completing the literature searches, titles and abstracts of the studies were screened by Yinan Zhao and Yanguo Xin, and any disagreement was resolved by discussion or, if necessary, adjudicated by Wenyu Hu.

### 2.2 Inclusion and exclusion criteria

The inclusion criteria were as follows: (a) NF155 was detected in serum, cerebrospinal fluid (CSF), or plasma exchange (PE) in patients with CIDP and control group; (b) investigation of the true‐positive (TP), false‐negative (FN) detection rate, while the true‐negative (TN) and false‐positive (FN) detection rate of NF155 were represented in CIDP and control group, respectively; (c) investigation of the association between NF155 and male incidence, the frequency of subacute disease, cerebral ataxia, sensory ataxia, and tremor; (d) investigation of the association between NF155 and frequency of good response to intravenous immunoglobulin (IVIg) treatment; and (e) investigation of the association between NF155 and frequency of the central involvement.

The following studies were considered ineligible: (a) studies without sufficient data to allow for extraction of frequencies for TP/FN and TN/FR or to allow for extraction of frequencies of the clinical features in NF155‐positive and NF155‐negative CIDP patients; (b) if the same patient cohort was reported in several studies, we used the most recent or complete cohorts and excluded case reports, letters, editors, reviews, and nonhuman animal model research; and (c) two independent reviewers identify the titles and abstracts of manuscripts, and those considered irrelevant were excluded.

### 2.3 Data extraction and study quality

Data extraction was performed independently by two authors using a standard form. The following data were extracted from each study: the basic information of the study (surname of the first author and year of publication, country of the procedure performed), study design, group assignment, sample type, detection method, number of patients, and frequency of autoantibodies detection, frequencies of the clinical features in NF155‐positive and NF155‐negative CIDP patients; Table 1). The following variables were extracted from each study using a standardized data extraction template: title, authors, year of publication, name of study cohort, geographic location, sample size, percentage of men, and frequency of autoantibodies detection.

**Table 1:** Characteristics of the included studies in the meta‐analysis

Study	Region	*n*	NF antibody	Autoantibody isotype	NF (+) patients	Male (female)	Age in years	Sample source	Method
Ogata et al., 2015	Japan	54	NF155	Predominant IgG4	13	8 (5)	42.4 ± 18.4	Serum	FC
Devaux et al., 2016	France	533	NF155	IgG4	38	8 (30)	31 (10–61)	Serum	ELISA
Querol et al., 2014	Spain	53	NF155	IgG4	2	2 (0)	34 (22–68)	Serum	ELISA
Mathey et al., 2017	Australia	44	NF155/NF−186	Predominant IgG4	3	2 (1)	42 ± 12.5	Serum	ELISA
Burnor et al., 2018	USA	40	NF155	Predominant IgG4	4	2 (2)	39 (13–56)	Serum	Cell‐based essay
Kawamura et al., 2013	Japan	16	NF155	–	4	3 (1)	37 (22–57)	Serum/CSF	ELISA/Cell‐based assay
Ng et al., 2012	Germany	119	NF155/NF−186	Predominant IgG4	5	3 (2)	62 (43–83)	Serum	ELISA/FC
Kadoya et al., 2016	Japan	191	NF155	IgG4	15	11 (4)	32 ± 15	Serum	ELISA/Cell‐based assay
Yan et al., 2014	Australia	141	NF155	IgG	32	–	–	Serum	ELISA
Doppler, Appeltshauser, Kramer et al., 2015; Doppler, Appeltshauser, Wilhelmi et al., 2015	Germany	35	NF155	IgG	0	–	–	serum/PE	ELISA/Binding assays/FC

Note

CSF: cerebrospinal fluid; ELISA: enzyme‐linked immunosorbent assay; FC: flow cytometry; NF: neurofascin; PE: plasma exchange

### 2.4 Quality assessment

Newcastle–Ottawa scale (NOS), a star system to determine the risk of bias of all included studies in a meta‐analysis (Stang, 2010), was applied in this literature. Total NOS score ranges from 0 to 9 stars, and higher scores stand for better quality. All included articles scored 5 or higher stars through the system (Table 2). The assessment procedure was performed individually by Wenyu Hu and Yanguo Xin.

**Table 2:** Newcastle–Ottawa scale for assessing the quality of the included studies in meta‐analysis

Study	Represent activeness of the exposed cohort	Selection of the nonexposed cohort	Ascertainment of exposure	Demonstration that outcome of interest was not present at start of study	Comparability of cohorts on the basis of the design or analyses	Assessment of outcome	Follow‐up long enough for outcome occur	Adequacy of follow‐up of cohorts	Quality score
Ogata et al., 2015	*	*	*	*	*	*	*	*	8
Devaux et al., 2016	*	*	*	*	**	*	–	–	7
Querol et al., 2014	*	*	*	*	*	*	–	–	6
Mathey et al., 2017	*	*	*	*	*	*	–	–	6
Burnor et al., 2018	*	*	*	*	*	*	–	–	6
Kawamura et al., 2013	*	*	*	*	*	*	–	–	6
Ng et al., 2012	*	*	*	*	*	*	–	–	6
Kadoya et al., 2016	*	*	*	*	**	*	–	–	7
Yan et al., 2014	*	*	*	*	*	–	–	–	5
Doppler, Appeltshauser, Kramer et al., 2015; Doppler, Appeltshauser, Wilhelmi et al., 2015	*	*	*	*	*	–	–	–	5

Note

Each asterisk represents compliance with the item on the evaluation table of Newcastle‐Ottawa scale. A maximum of 2 stars can be allotted in the comparability assessment, one star indicates either exposed and non‐exposed individuals is matched in the design for the most important factor, the second one for any additional factor.

### 2.5 Statistical analysis

The statistical analysis was performed independently by two authors according to recommendations from the PRISMA statement and the Cochrane handbook from the Cochrane Collaboration. Pooled odds ratios (OR) with 95% confidence intervals (CIs) were calculated for clinical features between anti‐NF155 antibody‐positive and ‐negative patients using a random effects model. Cochrane's *Q* test was used to analyze heterogeneity among the studies, and an *I* ^2^ calculation was used to measure the proportion of total variation in the estimates of outcomes; *I* ^2^ with *p* < 0.05 was used to denote statistical significance. Subgroup analyses were also conducted in our meta‐analysis. Meta‐analyses were performed using RevMan software version 5.3 provided by the Cochrane Collaboration. For prognostic analysis, the HRs were log‐transformed, and the standard errors of these log HRs were calculated from their 95% CIs in STATA 14.0 (StataCorp, College Station, TX, USA) and pooled analyses performed using meta commands. To assess for publication bias, we inspected funnel plots and performed the Egger's regression test and Begg's adjusted rank correlation test using the meta‐bias commands in STATA 14.0. All of the statistical tests were two‐sided, and a *p*‐value <0.05 was considered to be statistically significant.

## 3 RESULTS

### 3.1 Literature selection

**Figure 1:**
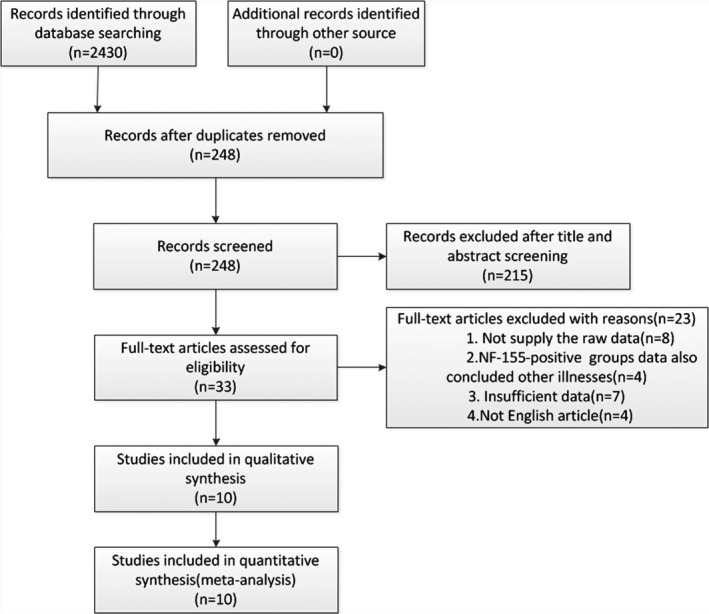
Flowchart of study selection

Database searches yielded 2,430 entries, of which 2,182 were excluded because of duplications, reviews, or irrelevance. Of the 248 publications, 215 studies were excluded through screening the titles, abstracts, publication types, and full texts; 23 studies were excluded because there were not sufficient data to allow for extraction of TP/FN and TN/FR frequencies or for extraction of the clinical features frequencies in NF155‐positive and ‐negative CIDP patients. Finally, we enrolled 10 trials for further analysis (Burnor et al., 2018; Devaux et al., 2016; Doppler, Appeltshauser, Kramer et al., 2015; Doppler, Appeltshauser, Wilhelmi et al., 2015; Kadoya et al., 2016; Kawamura et al., 2013; Mathey et al., 2017; Ng et al., 2012; Ogata et al., 2015; Querol et al., 2014; Yan et al., 2014). The PRISMA flow diagram for study selection is shown in Figure 1.

### 3.2 Diagnostic accuracy

**Figure 2:**
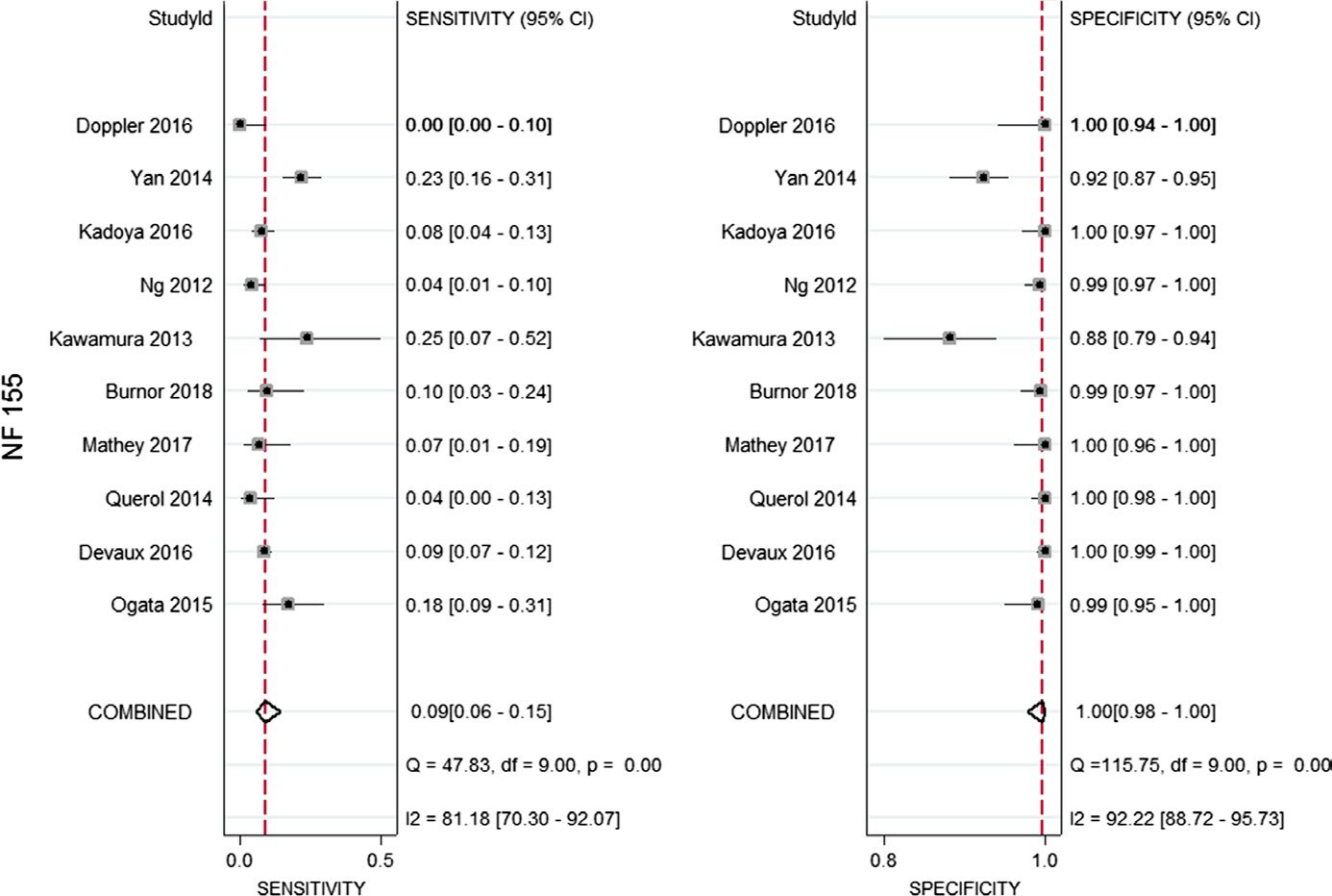
Forest plots of sensitivity and specificity for NF155 in CIDP patients

**Figure 3:**
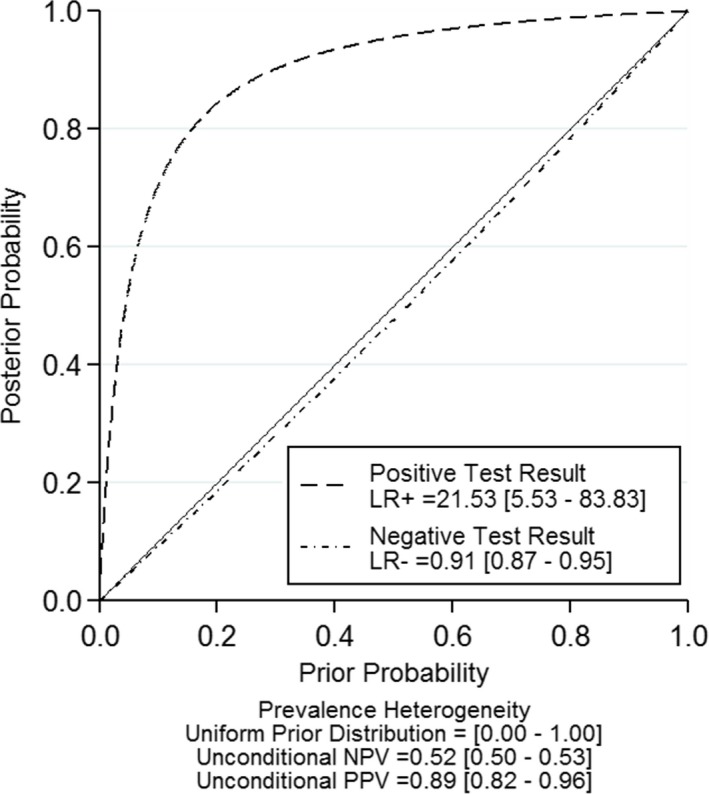
Post‐test probabilities of NF155 versus prior probabilities using summary likelihood ratios in CIDP

**Figure 4:**
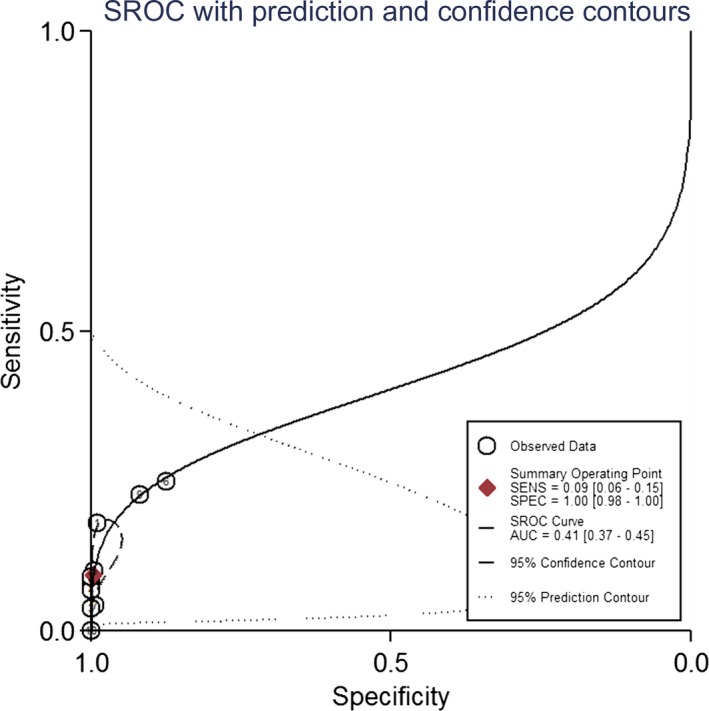
Summary receiver operating characteristics of NF155 in CIDP

**Figure 5:**
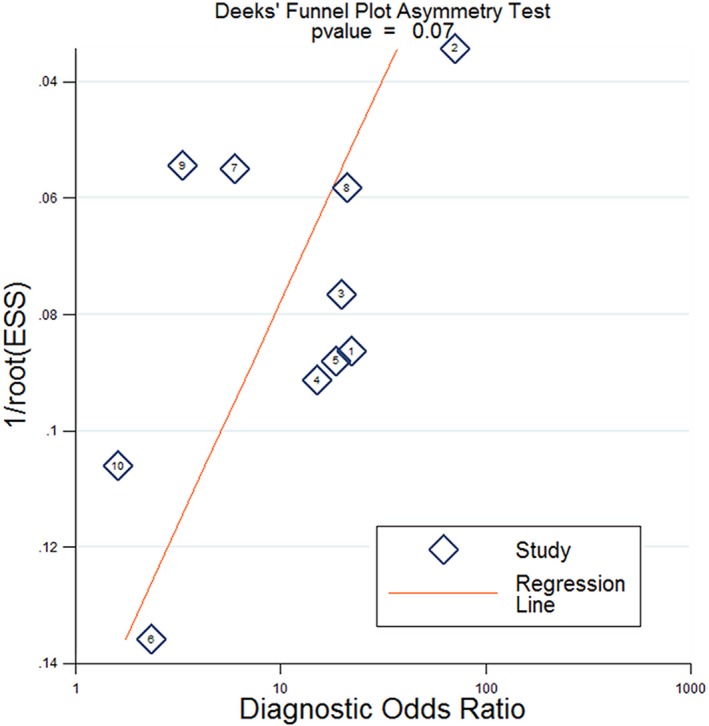
Publication bias test of the diagnostic meta‐analysis

The forest plots of SEN and SPE for NF155 are shown in Figure 2. The pooled SEN and SPE of the included studies of NF155 were 0.09 (95% CI: 0.06–0.15) and 1.00 (0.98–1.00); the pooled PLR, NLR, DOR, AUC, and their 95% confidence intervals were as follows: 21.5 (95% CI: 5.5– 83.8), 0.91 (95% CI: 0.87–0.95), 8.21 (95% CI: 3.57–18.89), and 0.41 (95% CI: 0.37–0.45; Figures 2,3 and 4, Table 3). There was no publication bias for the 10 studies included in this meta‐analysis (Figure 5).

**Table 3:** Diagnostic meta‐analysis of neurofascin in CIDP

	NF155	NF 186	CNTN1
Sensitivity (95% CI)	0.09 (0.06–0.15)	0.01 (0–0.05)	0.05 (0.03–0.08)
Specificity (95% CI)	1.00 (0.98–1.00)	1.00 (0.91–1.00)	1.00 (0.93–1.00)
PLR (95% CI)	21.5 (5.5–83.8)	5.2 (0.3–94.4)	26.3 (0.5–1,260.9)
NLR (95% CI)	0.91 (0.87–0.95)	0.99 (0.98–1.00)	0.96 (0.93–0.98)
DOR (95% CI)	8.21 (3.57–18.89)	0.86 (0.06–13.24)	4.63 (2.01–10.71)
AUC (95% CI)	0.41 (0.37–0.45)	0.10 (0.08–0.13)	0.17 (0.14–0.21)
Publication bias	0.07	0.74	0.21

Note

AUC: area under the curve; CI: confidence interval; CIDP: chronic inflammatory demyelinating polyneuropathy; DOR: diagnostic odds ratio; NLR: negative likelihood ratio; PLR: positive likelihood ratio.

### 3.3 Overall association of NF antibody detection with clinical features of CIDP

**Figure 6:**
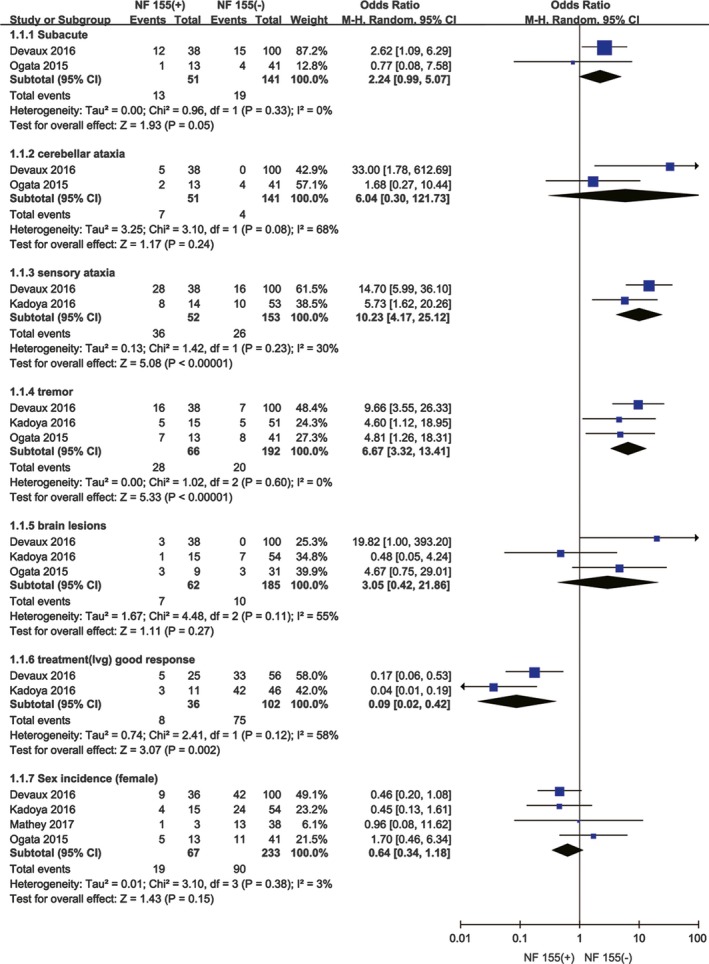
Forest plots of weighted mean difference (WMD) in NF155‐positive CIDP group and NF155‐negative CIDP group for clinical features. Horizontal lines are 95% confidence intervals

The evaluation mentioned above indicated that IgG4 anti‐NF155 could be a specific but not a sensitive parameter for CIDP. To identify the varieties of CIDP, we assessed the clinical features and the treatment response both in IgG4 anti‐NF155‐positive and ‐negative CIDP patients. We compared the sex incidence and occurrence rate of subacute, cerebellar ataxic, sensory ataxia and tremor, brain lesions, and IVIg treatment good response between the NF155‐positive and NF155‐negative CIDP patients. By comparing NF155‐positive CIDP patients with NF155‐negative CIDP patients, our meta‐analysis revealed that sensory ataxic occurrence rate (positive vs. negative: OR: 10.79, 95% CI: 5.24–22.22; *p* < 0.001), tremor occurrence rate (positive vs. negative: OR: 6.71, 95% CI: 3.37–13.39; *p* < 0.001), and the IVIg treatment good response rate (positive vs. negative: OR: 0.09, 95% CI: 0.02–0.42; *p* = 0.002) showed significant differences. In contrast, comparison of the sex incidence (positive vs. negative: OR: 1.70, 95% CI: 0.34–1.13; *p* = 0.12), subacute occurrence rate (positive vs. negative: OR: 2.17, 95% CI: 0.98–4.84; *p* = 0.06), cerebellar ataxia occurrence rate (positive vs. negative: OR: 6.04, 95% CI: 0.30–121.73; *p* = 0.24), and brain lesions (positive vs. negative: OR: 3.05, 95% CI: 0.42–21.86; *p* = 0.27) between NF155‐positive and ‐negative CIDP patients showed no significant differences. Random effect models were used in seven pairwise comparisons (sensory ataxia, anti‐NF155‐positive vs. ‐negative: *χ* ^2^ = 0.96, *p* = 0.33, *I* ^2^ = 0%; tremor, anti‐NF155‐positive vs. ‐negative: *χ* ^2^ = 1.02, *p* = 0.60, *I* ^2^ = 0%; sensory ataxia, positive vs. negative: *χ* ^2^ = 1.42, *p* = 0.23, *I* ^2^ = 30%; sex incidence, positive vs. negative: *χ* ^2^ = 3.10, *p* = 0.38, *I* ^2^ = 3%; cerebellar ataxia, positive vs. negative: *χ* ^2^ = 3.10, *p* = 0.08, *I* ^2^ = 68%; brain lesions, positive vs. negative: *χ* ^2^ = 3.10, *p* = 0.08, *I* ^2^ = 68%; IVIg treatment good response, positive vs. negative: *χ* ^2^ = 2.41, *p* = 0.12, *I* ^2^ = 58%; Table 4, Figure 6).

**Table 4:** Meta‐analysis of clinical features between NF155‐positive and ‐negative CIDP patients

	OR (95% CI)	*p* value	*p* for heterogeneity	*p* value for bias		Number of studies
				Begg's test	Egger's test	
Subacute	2.17 (0.98, 4.84)	0.06	0.33	1.00	–	2
Cerebellar ataxia	5.69 (1.60, 20.26)	0.007	0.08	1.00	–	2
Sensory ataxia	10.79 (5.24, 22.22)	<0.001	0.23	1.00	–	2
Tremor	6.71 (3.37, 13.39)	<0.001	0.60	0.296	0.064	3
Brain lesions	2.65 (0.95, 7.37)	0.06	0.11	1.00	0.801	3
Treatment (IVIg) good response	0.12 (0.05, 0.29)	<0.001	0.12	1.00	–	2
Sex incidence (female)	0.62 (0.34, 1.13)	0.12	0.38	0.308	0.505	4

Note

NF: neurofascin; CI: confidence interval; CIDP: chronic inflammatory demyelinating polyneuropathy; IVIg: intravenous immunoglobulin; OR: odds ratio.

### 3.4 Publication bias

According to the Deeks’ funnel plot asymmetry test, we found no significant correlation between study size and effect size or other evidence of publication bias (*p* = 0.07; Figure 5).

## 4 DISCUSSION

This report describes the first meta‐analysis study the diagnostic value of anti‐NF155 in CIDP patients. In our study, 10 published papers (Burnor et al., 2018; Devaux et al., 2016; Doppler, Appeltshauser, Kramer et al., 2015; Doppler, Appeltshauser, Wilhelmi et al., 2015; Kadoya et al., 2016; Kawamura et al., 2013; Mathey et al., 2017; Ng et al., 2012; Ogata et al., 2015; Querol et al., 2014; Yan et al., 2014) were collected to identify the diagnostic value of NF155 in CIDP patients; such parameters as sensitivity and specificity of the NF155 protein were calculated. We noticed that for CIDP diagnosis the pooled SEN, SPE, PLR, NLR, DOR, and AUC of NF155 were 0.09, 1.00, 21.5, 0.41, 8.21, and 0.91. This finding suggests that NF155 is a more specific marker protein for CIDP with questionable diagnostic value due to low sensitivity. Therefore, it may be more useful for defining clinical subsets of CIDP as an antibody against paranodal antigens.

The current meta‐analysis showed anti‐NF155 has low sensitivity for diagnostic value for CIDP patients; however, we found that the discrepancy of the anti‐NF155 detection frequencies from different studies may be due to different detection methods and inclusion criteria. As shown in Ogata's research (Ogata et al., 2015), anti‐human NF155 antibodies detected by specific cell‐based FCM assays were present in 18% of CIDP patients. The positivity rate of anti‐NF155 antibodies among CIDP patients in Ogata's study (18%) is much higher than others (2.5%, Ng et al., 2012 and 3.8%, Querol et al., 2014) using human recombinant NF155 as an antigen by ELISA (Ogata et al., 2015). Another reason for different detection rates of NF155 in CIDP patients may be inconsistent inclusion criteria. In Ogata's research (Ogata et al., 2015), they included the definite CIDP patients, who were adopted by EFNS/PNS diagnostic criteria and subsequently confirmed by electro‐diagnosis. However, several researchers used EFNS/PNS diagnostic criteria but did not mention that the cases enrolled in the research were probable or possible CIDP patients, and several studies did not describe the diagnostic criteria used in detail.

Consistent with a previous description (Querol et al., 2014), data from our meta‐analysis revealed that patients with positive anti‐NF155 antibody are more likely to be refractory to IVIg treatment. The mechanism of the poor response to IVIg treatment in anti‐NF155 antibody‐positive CIDP may be that IVIg is inhibitory to the complement pathway (Sudo, Yamaguchi, Spath, Matsumoto‐Morita, Ong, & Shahrizaila, 2014; Zhang, Lopez, Li, Mehta, Griffin, & Schnaar, 2004); however, an IgG subclass of the studies included in our analysis was mainly IgG4, which have a low affinity for Fc receptors and complement. In our meta‐analysis, anti‐NF155‐positive patients presented with more severe sensory ataxia and tremor involvement, which is rarely seen in anti‐NF155‐negative patients. The mechanism of marked sensory ataxia and tremor in anti‐NF155‐positive CIDP patients is still unclear. In several studies, researchers used the sera from anti‐NF155‐positive CIDP patients stain mouse teased sciatic nerve fibers; these studies found that anti‐NF155 antibody bound specifically to paranode regions of peripheral nerves, indicating that the paranode might be the main target. At the paranode, glial NF155 and the complex of axonal contactin‐1 and Caspr1 form a septate‐like junction that anchors myelin loops to the axon. A previous study (Devaux et al., 2016) speculated that blocking the anti‐CNTN1 antibodies through the antigen may preferentially affect the sensory axon paranode. A recent report (Doppler, Appeltshauser, Wilhelmi, Villmann, Dib‐Hajj, & Waxman, 2015) indicated that patients with anti‐CNTN1 IgG4 antibodies showed specific paranodal alterations in dermal nerve biopsies. In a NF155 mouse model, myelinating glia‐specific ablation decreased conduction velocities in peripheral nerves, indicating that IgG4 anti‐NF155 antibodies may block interactions between NF155 and Caspr1/contactin‐1 leading to conduction failure (Pillai et al., 2009). Based on the accumulating evidence and the results of our meta‐analysis, we speculate that disconnection of NF155 and Caspr1/contactin‐1 may induce decreased conduction velocities and lead to the distinguishing features described above.

Three studies compared the brain MRI lesions of patients with positive or negative anti‐NF155 antibody (Miura et al., 2015; Querol et al., 2013; Yan et al., 2014). Demyelination was more likely to occur in anti‐NF155‐positive patients, despite the lack of statistical significance in the current meta‐analysis, suggesting an association between the anti‐NF155 and central nervous system involvement. Thus, anti‐NF155 is a probable predictive biomarker for CNS involvement in patients with CIDP. However, the mechanism by which anti‐NF155 antibody preferentially affects both the PNS and the CNS remains unclear. In the future, more large‐scale studies are required to clarify this question.

NF186 is the transmembrane protein found in the axon that functions as a cell adhesion molecule together with NrCAM comprising the nodal complex (Davis, 1996; Tait, Gunn‐Moore, Collinson, Huang, Lubetzki, & Pedraza, 2000). In a NF186 mouse model, neuron‐specific ablation decreased conduction velocities in peripheral nerves (Thaxton, Pillai, Pribisko, Dupree, & Bhat, 2011). We also tested the association between nodes protein NF186 together with another paranode protein contactin‐1 and CIDP patients. Studies related to NF‐186 (Delmont, Manso, Querol, Cortese, Berardinelli, & Lozza, 2017; Devaux, 2012; Mathey et al., 2017; Ng et al., 2012; Notturno, Di Febo, Yuki, Fernandez Rodriguez, Corti, & Nobile‐Orazio, 2014; Ogata et al., 2015; Querol et al., 2014) and studies related to CNTN‐1 (Doppler, Appeltshauser, Kramer et al., 2015; Doppler, Appeltshauser, Villmann et al., 2015; Doppler, Appeltshauser, Villmann, Martin, Peles, & Kramer, 2016; Mathey et al., 2017; Miura, Devaux, Fukami, Manso, Belghazi, & Wong, 2015; Querol, Nogales‐Gadea, Rojas‐Garcia, Martinez‐Hernandez, Diaz‐Manera, & Suarez‐Calvet, 2013) were pooled and analyzed (Table 3). The results indicate that similar to NF155, both anti‐NF‐186 and anti‐CNTN1 have questionable diagnostic value due to low sensitivity. However, these antibodies may be useful as more specific marker proteins to clinically define subsets of CIDP.

Three studies focused on the clinical features on anti‐CNTN1‐positive CIDP patients. All of the studies displayed an acute onset and a rapid progressive disease course. All patients with anti‐CNTN1 IgG4 antibodies showed sensory ataxia (Doppler, Appeltshauser, Kramer et al., 2015; Doppler, Appeltshauser, Villmann et al., 2015; Miura et al., 2015) and were poorly responsive to IVIg treatment but sensitive to steroid treatments (Miura et al., 2015; Querol et al., 2013). Compared to anti‐NF155, several studies concluded that CIDP patients lack reactivity to anti‐NF186 antibody (Devaux et al., 2016; Ng et al., 2012; Ogata et al., 2015), whereas a recent study reported that anti‐186 antibody was found with 2% prevalence in CIDP patients (Delmont et al., 2017), and within five anti‐186 antibody‐positive patients, conduction block and cranial nerves were involved in two patients; 75% of the anti‐NF186‐positive CIDP patients responded well to both IVIg and steroid treatments. The discrepancy may be caused by low prevalence of anti‐NF186 antibodies. More international groups conducting larger studies are expected to investigate both the frequencies of the anti‐NF186 and anti‐CNTN1 autoantibodies, clinical features, and treatment responses of CIDP patients in different populations and countries.

There are several limitations to our study. First, the studies included in our meta‐analysis examined 10 and 2,797 patients, meaning that there were not sufficient data in the subgroup analyses. Second, the diagnostic criteria for CIDP and the anti‐NF155 antibody detection methods lack uniformity, which may affect the validity of NF155 as a predictive marker to define CIDP subclasses. Finally, the significant heterogeneity among these studies might be observed because the patients included in each study might come from different races and experimental methods, and the year published varied. Based on the limitations of the present study, larger sample sizes and more well‐designed multicenter trials are suggested.

## CONFLICT OF INTEREST

None declared.

## References

[brb31115-bib-0001] Burnor, E. , Yang, L. , Zhou, H. , Patterson, K. R. , Quinn, C. , Reilly, M. M. , … Lancaster, E. (2018). Neurofascin antibodies in autoimmune, genetic, and idiopathic neuropathies. Neurology, 90, e31–e38. 10.1212/WNL.0000000000004773. 29187518 PMC5754648

[brb31115-bib-0002] Charles , P. , Tait , S. , Faivre‐Sarrailh , C. , Barbin , G. , Gunn‐Moore , F. , Denisenko‐Nehrbass , N. , … Lubetzki , C. (2002). Neurofascin is a glial receptor for the paranodin/Caspr‐contactin axonal complex at the axoglial junction. Current Biology, 12, 217–220. 10.1016/S0960-9822(01)00680-7. 11839274

[brb31115-bib-0003] Davis, J. Q. (1996). Molecular composition of the node of Ranvier: Identification of ankyrin‐ binding cell adhesion molecules neurofascin (mucin+/third FNIII domain‐ ) and NrCAM at nodal axon segments. The Journal of Cell Biology, 135, 1355–1367. 10.1083/jcb.135.5.1355. 8947556 PMC2121080

[brb31115-bib-0004] Delmont, E. , Manso, C. , Querol, L. , Cortese, A. , Berardinelli, A. , Lozza, A. , … Devaux, J. J. (2017). Autoantibodies to nodal isoforms of neurofascin in chronic inflammatory demyelinating polyneuropathy. Brain, 140, 1851–1858. 10.1093/brain/awx124. 28575198

[brb31115-bib-0005] Devaux, J. J. (2012). Antibodies to gliomedin cause peripheral demyelinating neuropathy and the dismantling of the nodes of Ranvier. American Journal of Pathology, 181, 1402–1413. 10.1016/j.ajpath.2012.06.034. 22885108 PMC5691341

[brb31115-bib-0006] Devaux, J. J. , Miura, Y. , Fukami, Y. , Inoue, T. , Manso, C. , Belghazi, M. , … Yuki, N. (2016). Neurofascin‐155 IgG4 in chronic inflammatory demyelinating polyneuropathy. Neurology, 86, 800–807. 10.1212/WNL.0000000000002418. 26843559 PMC4793783

[brb31115-bib-0007] Doppler, K. , Appeltshauser, L. , Kramer, H. H. , Ng, J. K. , Meinl, E. , Villmann, C. , … Sommer, C. (2015). Contactin‐1 and Neurofascin‐155/‐186 are not targets of auto‐antibodies in multifocal motor neuropathy. PLoS One, 10, e0134274 10.1371/journal.pone.0134274. 26218529 PMC4517860

[brb31115-bib-0008] Doppler, K. , Appeltshauser, L. , Villmann, C. , Martin, C. , Peles, E. , Kramer, H. H. , … Sommer, C. (2016). Auto‐antibodies to contactin‐associated protein 1 (Caspr) in two patients with painful inflammatory neuropathy. Brain, 139, 2617–2630. 10.1093/brain/aww189. 27474220

[brb31115-bib-0009] Doppler, K. , Appeltshauser, L. , Wilhelmi, K. , Villmann, C. , Dib‐Hajj, S. D. , Waxman, S. G. , … Sommer, C. (2015). Destruction of paranodal architecture in inflammatory neuropathy with anti‐contactin‐1 autoantibodies. Journal of Neurology, Neurosurgery and Psychiatry, 86, 720–728. 10.1136/jnnp-2014-309916. 25694474

[brb31115-bib-0010] Hughes, R. A. , Bouche, P. , Cornblath, D. R. , Evers, E. , Hadden, R. D. , Hahn, A. , … van Schaik, I. N. (2006). European Federation of Neurological Societies/Peripheral Nerve Society guideline on management of chronic inflammatory demyelinating polyradiculoneuropathy: Report of a joint task force of the European Federation of Neurological Societies and the Peripheral Nerve Society. European Journal of Neurology, 13, 326–332. 10.1111/j.1468-1331.2006.01278.x. 16643309

[brb31115-bib-0011] Kadoya, M. , Kaida, K. , Koike, H. , Takazaki, H. , Ogata, H. , Moriguchi, K. , … Ikewaki, K. (2016). IgG4 anti‐neurofascin155 antibodies in chronic inflammatory demyelinating polyradiculoneuropathy: Clinical significance and diagnostic utility of a conventional assay. Journal of Neuroimmunology, 301, 16–22. 10.1016/j.jneuroim.2016.10.013. 27852440

[brb31115-bib-0012] Kawamura, N. , Yamasaki, R. , Yonekawa, T. , Matsushita, T. , Kusunoki, S. , Nagayama, S. , … Kira, J. (2013). Anti‐neurofascin antibody in patients with combined central and peripheral demyelination. Neurology, 81, 714–722. 10.1212/WNL.0b013e3182a1aa9c. 23884033

[brb31115-bib-0013] Latov, N. (2014). Diagnosis and treatment of chronic acquired demyelinating polyneuropathies. Nature Reviews Neurology, 10, 435–446. 10.1038/nrneurol.2014.117. 24980070

[brb31115-bib-0014] Mathey, E. K. , Garg, N. , Park, S. B. , Nguyen, T. , Baker, S. , Yuki, N. , … Kiernan, M. C. (2017). Autoantibody responses to nodal and paranodal antigens in chronic inflammatory neuropathies. Journal of Neuroimmunology, 309, 41–46. 10.1016/j.jneuroim.2017.05.002. 28601286

[brb31115-bib-0015] Miura , Y. , Devaux , J. J. , Fukami , Y. , Manso , C. , Belghazi , M. , Wong , A. H. , …CNTN1‐CIDP Study Group (2015). Contactin 1 IgG4 associates to chronic inflammatory demyelinating polyneuropathy with sensory ataxia. Brain, 138, 1484–1491. 10.1093/brain/awv054. 25808373 PMC4614146

[brb31115-bib-0016] Ng, J. K. , Malotka, J. , Kawakami, N. , Derfuss, T. , Khademi, M. , Olsson, T. , … Meinl, E. (2012). Neurofascin as a target for autoantibodies in peripheral neuropathies. Neurology, 79, 2241–2248. 10.1212/WNL.0b013e31827689ad. 23100406 PMC3542349

[brb31115-bib-0017] Notturno, F. , Di Febo, T. , Yuki, N. , Fernandez Rodriguez, B. M. , Corti, D. , Nobile‐Orazio, E. , … Uncini, A. (2014). Autoantibodies to neurofascin‐186 and gliomedin in multifocal motor neuropathy. Journal of Neuroimmunology, 276, 207–212. 10.1016/j.jneuroim.2014.09.001. 25283719

[brb31115-bib-0018] Ogata, H. , Yamasaki, R. , Hiwatashi, A. , Oka, N. , Kawamura, N. , Matsuse, D. , … Kira, J. (2015). Characterization of IgG4 anti‐neurofascin 155 antibody‐positive polyneuropathy. Annals of Clinical and Translational Neurology, 2, 960–971. 10.1002/acn3.248. 26478896 PMC4603379

[brb31115-bib-0019] Pillai, A. M. , Thaxton, C. , Pribisko, A. L. , Cheng, J. G. , Dupree, J. L. , & Bhat, M. A. (2009). Spatiotemporal ablation of myelinating glia‐specific neurofascin (Nfasc NF155) in mice reveals gradual loss of paranodal axoglial junctions and concomitant disorganization of axonal domains. Journal of Neuroscience Research, 87, 1773–1793. 10.1002/jnr.22015. 19185024 PMC2837286

[brb31115-bib-0020] Querol, L. , Nogales‐Gadea, G. , Rojas‐Garcia, R. , Diaz‐Manera, J. , Pardo, J. , Ortega‐Moreno, A. , … Illa, I. (2014). Neurofascin IgG4 antibodies in CIDP associate with disabling tremor and poor response to IVIg. Neurology, 82, 879–886. 10.1212/WNL.0000000000000205. 24523485 PMC3959751

[brb31115-bib-0021] Querol, L. , Nogales‐Gadea, G. , Rojas‐Garcia, R. , Martinez‐Hernandez, E. , Diaz‐Manera, J. , Suarez‐Calvet, X. , … Illa, I. (2013). Antibodies to contactin‐1 in chronic inflammatory demyelinating polyneuropathy. Annals of Neurology, 73, 370–380. 10.1002/ana.23794. 23280477

[brb31115-bib-0022] Stang, A. (2010). Critical evaluation of the Newcastle‐Ottawa scale for the assessment of the quality of nonrandomized studies in meta‐analyses. European Journal of Epidemiology, 25, 603–605. 10.1007/s10654-010-9491-z. 20652370

[brb31115-bib-0023] Sudo, M. , Yamaguchi, Y. , Spath, P. J. , Matsumoto‐Morita, K. , Ong, B. K. , Shahrizaila, N. , & Yuki, N. (2014). Different IVIG glycoforms affect in vitro inhibition of anti‐ganglioside antibody‐mediated complement deposition. PLoS One, 9, e107772 10.1371/journal.pone.0107772. 25259950 PMC4178036

[brb31115-bib-0024] Tait, S. , Gunn‐Moore, F. , Collinson, J. M. , Huang, J. , Lubetzki, C. , Pedraza, L. , … Brophy, P. J. (2000). An oligodendrocyte cell adhesion molecule at the site of assembly of the paranodal axo‐glial junction. The Journal of Cell Biology, 150, 657–666. 10.1083/jcb.150.3.657. 10931875 PMC2175192

[brb31115-bib-0025] Thaxton, C. , Pillai, A. M. , Pribisko, A. L. , Dupree, J. L. , & Bhat, M. A. (2011). Nodes of Ranvier act as barriers to restrict invasion of flanking paranodal domains in myelinated axons. Neuron, 69, 244–257. 10.1016/j.neuron.2010.12.016. 21262464 PMC3035172

[brb31115-bib-0026] Van den Bergh, P. Y. , Hadden, R. D. , Bouche, P. , Cornblath, D. R. , Hahn, A. , Illa, I. , … Peripheral Nerve, S. (2010). European Federation of Neurological Societies/Peripheral Nerve Society guideline on management of chronic inflammatory demyelinating polyradiculoneuropathy: Report of a joint task force of the European Federation of Neurological Societies and the Peripheral Nerve Society ‐ first revision. European Journal of Neurology, 17, 356–363. 10.1111/j.1468-1331.2009.02930.x. 20456730

[brb31115-bib-0027] Yan, W. , Nguyen, T. , Yuki, N. , Ji, Q. , Yiannikas, C. , Pollard, J. D. , & Mathey, E. K. (2014). Antibodies to neurofascin exacerbate adoptive transfer experimental autoimmune neuritis. Journal of Neuroimmunology, 277, 13–17. 10.1016/j.jneuroim.2014.09.012. 25262157

[brb31115-bib-0028] Zhang, G. , Lopez, P. H. , Li, C. Y. , Mehta, N. R. , Griffin, J. W. , Schnaar, R. L. , & Sheikh, K. A. (2004). Anti‐ganglioside antibody‐mediated neuronal cytotoxicity and its protection by intravenous immunoglobulin: Implications for immune neuropathies. Brain, 127, 1085–1100. 10.1093/brain/awh127. 14985267

